# Papillorenal Syndrome-Causing Missense Mutations in *PAX2*/*Pax2* Result in Hypomorphic Alleles in Mouse and Human

**DOI:** 10.1371/journal.pgen.1000870

**Published:** 2010-03-05

**Authors:** Ramakrishna P. Alur, Camasamudram Vijayasarathy, Jacob D. Brown, Mohit Mehtani, Ighovie F. Onojafe, Yuri V. Sergeev, Elangovan Boobalan, MaryPat Jones, Ke Tang, Haiquan Liu, Chun-hong Xia, Xiaohua Gong, Brian P. Brooks

**Affiliations:** 1 Ophthalmic Genetics and Visual Function Branch, National Eye Institute, National Institutes of Health, Department of Health and Human Services, Bethesda, Maryland, United States of America; 2 Section for Translational Research in Retinal and Macular Degeneration, National Institute on Deafness and Other Communication Disorders, National Institutes of Health, Department of Health and Human Services, Bethesda, Maryland, United States of America; 3 Department of Biochemistry, Molecular and Cellular Biology, Georgetown University School of Medicine, Washington, D.C., United States of America; 4 National Human Genome Research Institute, National Institutes of Health, Department of Health and Human Services, Bethesda, Maryland, United States of America; 5 Department of Molecular and Cellular Biology, Baylor College of Medicine, Houston, Texas, United States of America; 6 School of Optometry and Vision Science Program, University of California Berkeley, Berkeley, California, United States of America; University of Michigan, United States of America

## Abstract

Papillorenal syndrome (PRS, also known as renal-coloboma syndrome) is an autosomal dominant disease characterized by potentially-blinding congenital optic nerve excavation and congenital kidney abnormalities. Many patients with PRS have mutations in the paired box transcription factor gene, *PAX2*. Although most mutations in *PAX2* are predicted to result in complete loss of one allele's function, three missense mutations have been reported, raising the possibility that more subtle alterations in PAX2 function may be disease-causing. To date, the molecular behaviors of these mutations have not been explored. We describe a novel mouse model of PRS due to a missense mutation in a highly-conserved threonine residue in the paired domain of *Pax2* (p.T74A) that recapitulates the ocular and kidney findings of patients. This mutation is in the *Pax2* paired domain at the same location as two human missense mutations. We show that all three missense mutations disrupt potentially critical hydrogen bonds in atomic models and result in reduced Pax2 transactivation, but do not affect nuclear localization, steady state mRNA levels, or the ability of Pax2 to bind its DNA consensus sequence. Moreover, these mutations show reduced steady-state levels of Pax2 protein *in vitro* and (for p.T74A) *in vivo*, likely by reducing protein stability. These results suggest that hypomorphic alleles of *PAX2/Pax2* can lead to significant disease in humans and mice.

## Author Summary

Congenital ocular malformations affecting the optic nerve are an important cause of childhood blindness. The papillorenal syndrome (PRS) is an autosomal dominant disorder that causes congenital optic nerve and kidney abnormalities, which may result in legal blindness and renal failure, respectively. Many cases of PRS are caused by mutations in the paired-box transcription factor *PAX2*. In this paper, we describe a novel mouse model of this human disease caused by a missense mutation in the *Pax2* gene at the same position of one of the few disease-causing missense mutations in humans. We characterize the ocular and non-ocular phenotypes of this mouse and model the effect that murine and human *Pax2/PAX2* mutations have on protein structure. We also experimentally test the effect these missense mutations have on protein localization, transactivation, and DNA binding, concluding that all three reduce steady-state levels of protein *in vitro* and (in p.T74A) *in vivo* by reducing protein stability. This work will help us better understand the pathophysiology of PRS and to dissect the molecular interactions important in normal PAX2 function.

## Introduction

Papillorenal syndrome (PRS, OMIM#120330, renal-coloboma syndrome) is an autosomal dominant condition characterized by congenital anomalies of the optic nerve and kidney [1]–[3]. Kidney abnormalities range from aplasia or hypoplasia to cystic and dysplastic changes [4]–[15]. These abnormalities, coupled with the vesico-ureteral reflux observed in some patients, may lead to renal failure [7],[8],[16],[17]. Ocular abnormalities range from asymptomatic differences in retinal blood vessel patterning and optic nerve pits to blinding congenital excavations of the optic nerve head [5]. Although the excavation and vascular abnormalities can be quite subtle [10], other cases are reminiscent of the morning glory anomaly or may be mistaken as normal-tension glaucoma. Additional ocular features include the absence or hypoplasia of the central retinal artery, foveal hypoplasia, and anomalous retinal and choroidal perfusion leading to retinal thinning and visual field deficits [7]. Some patients with PRS also have high frequency hearing loss [2],[4],[6],[10],[17],[18]. Schimmenti *et al.* have suggested that Chiari 1 malformations and other CNS malformations may also be an uncommon feature of this syndrome [17],[19]. Germline mosaicism has been reported [9].

Many patients with PRS have a mutation in the *PAX2* gene, a member of the paired box family of transcription factor genes [4],[6], that is normally expressed in the developing kidney, optic cup, otic vesicle and midbrain-hindbrain boundary [20]–[23]. To date, the vast majority of pathologic *PAX2* mutations are predicted to cause complete loss of function of one allele (haploinsufficiency) [4]–[6],[9],[10],[13],[14],[18],[19]. The existing mouse models of *PAX2* haploinsufficiency appropriately reflect the ocular, urogenital, and otic abnormalities noted in human patients [22]–[26].

A few patients with PRS, however, have been reported to have missense mutations, two of which cluster in the paired domain of the protein [15],[16]. The molecular mechanism by which these mutations lead to disease has remained unexplored. We have identified and characterized a novel mouse model of PRS in which a paired domain missense mutation occurs at the same position as in some humans with the disease. Furthermore, we have characterized the molecular basis for this mouse mutation, as well as for the paired domain missense mutations reported in humans. We show that the mutant mice recapitulate the ocular and kidney phenotypes of patients with PRS. We model the effect of these mutations on Pax2 structure *in silico* and demonstrate that these mutant proteins are expressed *in vitro* and *in vivo* at lower steady-state levels than wild-type protein and that this leads to a commensurate reduction in *Pax2* transactivation and protein stability *in vitro*. Furthermore, we observe that these mutations do not appear to affect nuclear localization, the steady-state levels of *Pax2* mRNA or the ability of these proteins to bind a *PAX2* consensus sequence *in vitro*. These combined results argue that patients with these missense mutations in *PAX2* likely develop PRS because of the hypomorphic nature of these alleles and that their residual function is not sufficient to prevent significant ocular and renal disease.

## Results

### Identification of mouse mutant

**Figure 1:**
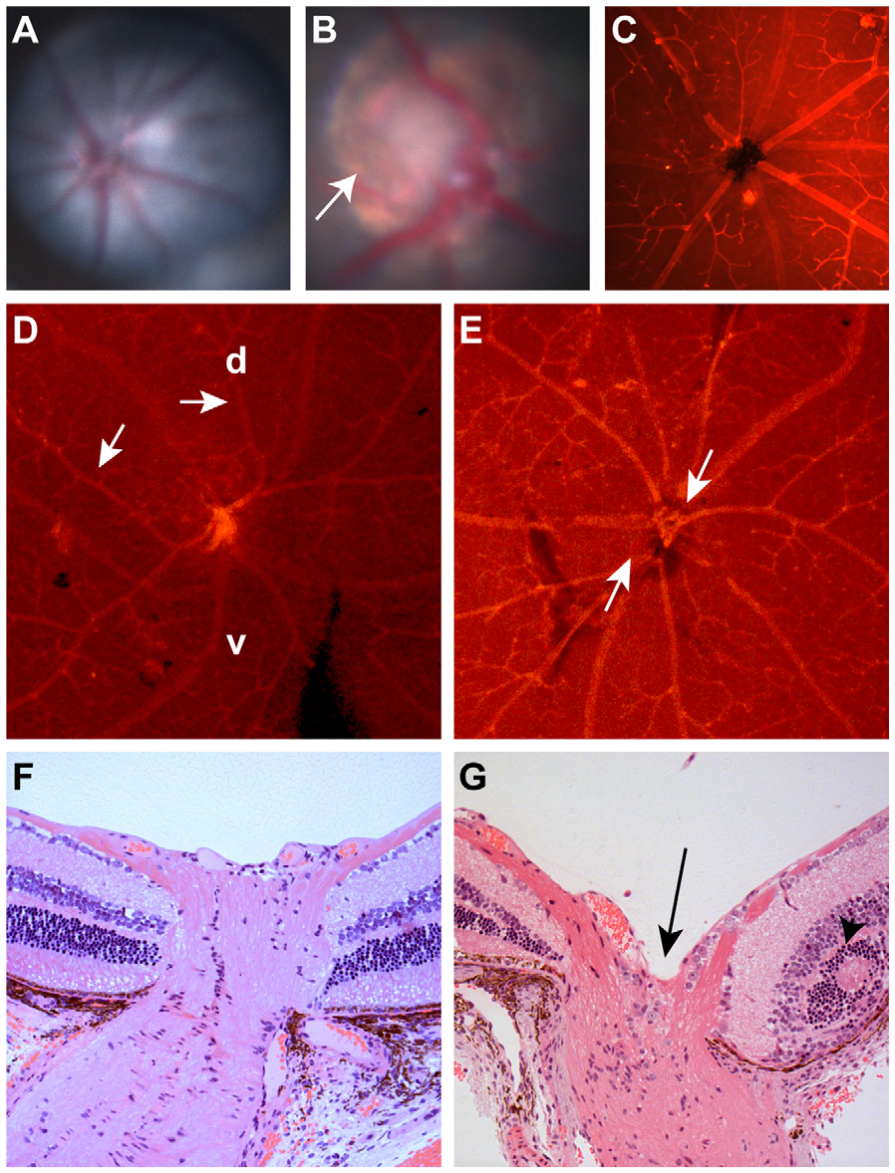
Clinical ocular phenotype in C57BL/6-*Pax2^+/A220G^* mice compared to wild-type, C57BL/6 mice. (A) Fundus photograph of C57BL/6 mouse showing normal optic nerve and radial pattern of retinal blood vessels. (B) Fundus photograph of C57BL/6-*Pax2^+/A220G^* mouse showing congenital excavation of the optic nerve head with peripapillary pigment changes (arrow). (C) Lectin immunofluorescence of wild-type C57BL/6 mouse showing normal, radial vessel patterning. (D,E) Lectin immunofluorescence of C57BL/6-*Pax2^+/A220G^* mice showing abnormal vascular patterning, including curving of vessels towards the dorsal retina (D, arrows, d = dorsal, v = ventral) and separation of the central retinal vascular trunks (E, arrows). Histologic section of a *Pax2^+/+^* (F) and a *Pax2^+/A220G^* (G) mouse eye through the optic nerve and peripapillary retina showing abnormal excavation of the optic nerve (G, arrow) and retinal rosette formation (G, arrowhead). Remnants of the *tunica vasculosis lentis* and mild extension of the retinal pigment epithelium were variably noted in histopathology from other *Pax2^A220G/+^* eyes (data not shown).

During our ENU mutagenesis screen of C57BL/6 mice, we discovered a line of mice that exhibited congenital excavation of the optic nerve head and abnormal patterning of retinal blood vessels (Figure 1). This excavation was confirmed on histologic sections, which also show abnormalities in retinal lamination, such as rosette formation. This phenotype was transmitted as an autosomal dominant trait with complete penetrance on the native C57BL/6 background. Mapping was performed by mating affected C57BL/6 mice of either gender to C3H/HeJ mice to produce G1 progeny. Affected mice were then backcrossed to wild-type C57BL/6 mice. Assaying twelve of these affected G2 progeny for homozygosity of C57BL/6 markers revealed zero recombinants at chromosome markers D19Mit120 and D19Mit17.1. Of the 61 G1 animals and 122 G2 animals ascertained, 10 animals (16%, 7 males, 3 females) and 31animals (25%, 9 males, 22 females), respectively, met criteria for “affected” status; this finding suggests reduced penetrance for the optic nerve phenotype on the C3H/HeJ genetic background.

**Figure 2:**
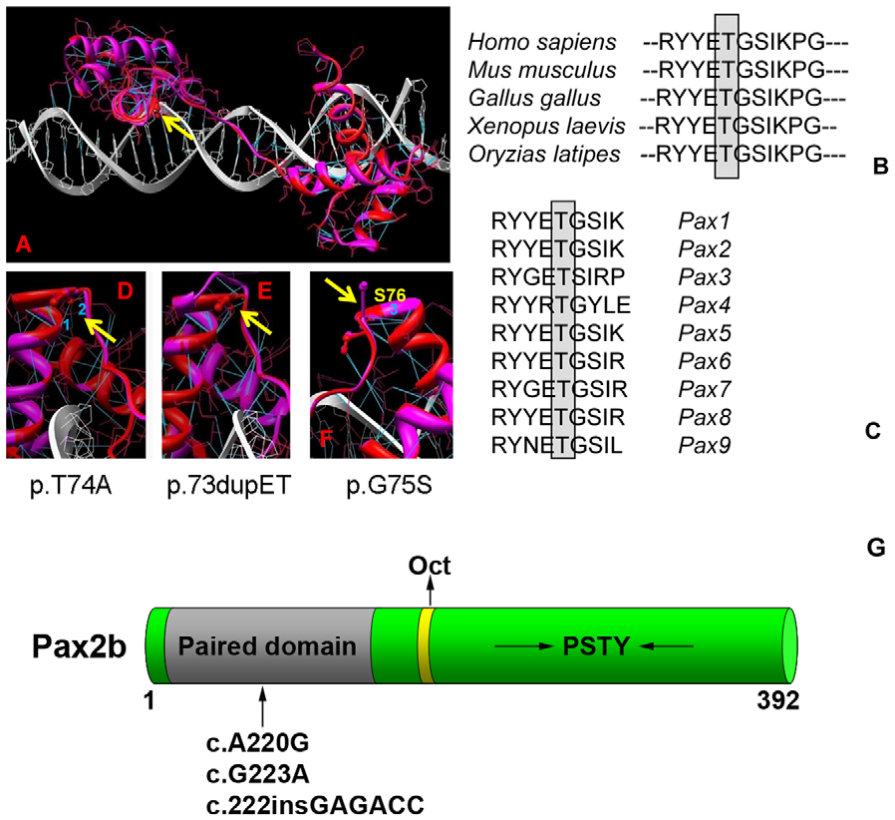
Homology modeling of the wild-type and mutant Pax2 paired domain-DNA complex. The paired domain of wild-type Pax2 domain DNA are represented by red and white ribbons, respectively, and their corresponding atomic structures are shown by red and white bonds (A). Hydrogen bonds are shown in blue. Threonine 74 in the mouse protein sequence (equivalent to T75A in human) is absolutely conserved across several species (B) and across all known murine Pax-family members (C). Fragments of the Pax2 paired domain–DNA complex modified by the mutations T74A, dup73ET and G75S are shown on (D–F), respectively. Hydrogen bonds presented in the wild type protein that are broken by the mutation T74A are labeled as 1 and 2 for (D) and by the mutation G75S is labeled as 3 (F). Yellow arrows indicate the location of mutations in Pax2 paired domain. A schematic of the Pax2b protein modified from Lechner *et al.* [32] showing the paired domain (gray), the octapeptide (Oct) domain (yellow), and the C-terminus, which is rich in proline, serine, threonine and tyrosine (PSTY) residues (G). Numbers indicate amino acid position. The arrow denotes the approximate position of the three mutations studied.

The paired-box transcription factor gene, *Pax2*, was noted to be within the critical interval on chromosome 19. Because mutation of *PAX2* in humans is known to result in congenital optic nerve abnormalities, we considered it an excellent candidate gene. Sequencing of the coding exons and the intron-exon boundaries of *Pax2* revealed a heterozygous c.A220G sequence change, predicted to change threonine 74 to an alanine at the protein level. This threonine is invariantly conserved in *Pax2* across several vertebrate species (human, mouse, chicken, frog, Medaka fish) and in all murine members of the paired-box family (*Pax1* through *Pax9*) (Figure 2B and 2C).

### Structural characterization of wild-type and mutant Pax2 proteins

To better understand the role this mutation may be playing in Pax2 protein structure, we created an atomic model of the Pax2 paired domain-DNA hetero-complex (Figure 2A). Because the modeling of Pax2 was performed using structural information from the homeodomain of PAX6 as a guide, all interactions we described should be considered predicted. The Pax2 paired domain contains 2 similar globular protein sub-domains, known as N (residues 16–74) and C (residues 88–148) subdomains, linked by the extended 12-residue polypeptide chain (residues 75–87) similar to that of PAX6 paired domain [27]. The Pax2 N-subdomain includes an anti-parallel β-hairpin (residues 16–27) and 3 α-helices, α1 (residues 33–46), α2 (residues 49–57), and α3 (residues 58–74), folded like a homeodomain. The C-subdomain also include 3 α-helices, α4 (residues 88–105), α5 (residues 109–120) and α6 (residues 131–146), related by approximate 2-fold symmetry to N-domain helices. Although the C-subdomain is involved in protein-DNA interaction, the N-subdomain plays a dominant role in DNA binding of the intact PAX6 paired domain [27]. From a previous crystallographic study, it was suggested that conserved residues at the end of α3-helix help to fix the position of the extended linker by conservation of hydrogen bonds and hydrophobic interactions [28]. In addition, the α3-helix, known as a recognition helix, is a part of a helix-turn-helix (HTH) motif, involving helices α2 and α3. All these data suggest that the properties of the conserved sequence at the end of the α3-helix and at the beginning of the polypeptide linker are important to maintain the paired domain-DNA interactions.

In the mutant T74A, the polar threonine is replaced by hydrophobic alanine residue breaking 2 hydrogen bonds (H-bonds 1 and 2 at corresponding distances 2.8 Å and 3.1 Å) present in wild type protein as shown in Figure 2D. In wild type protein these H-bonds are connecting γ-oxygen of threonine 74 to a backbone oxygen of residue R70 (H-bond 1) and a main chain nitrogen of serine 76 (H-bond 2). Missense mutation T74A disrupts both bonds (Figure 2D). Although Pax2 is known to be phosphorylated by kinases such the c-Jun N-terminal kinase (JNK) [29],[30], i*n silico* analysis of the *Pax2* protein sequence using two different programs (http://www.cbs.dtu.dk/services/NetPhos/ and http://scansite.mit.edu/motifscan_seq.phtml) predicted that threonine 74 is not a likely site for phosphorylation.

### Characterization of mouse mutant

Congenital optic nerve excavation co-segregated with the *Pax2^A220G^* allele 100% of the time in over 100 mice analyzed, indicating complete penetrance for this phenotype on the C57BL/6 background. Of 31 offspring of a *Pax2^A220G/+^* x *Pax2^A220G/+^* mating, 22 (71%) were affected, *Pax2^A220G/+^* and 9 (29%) were unaffected, *Pax2^+/+^*. No *Pax2^A220G/A220G^* mice were observed, which statistically deviates from the expected ratios of 1:2:1 homozygotes to heterozygotes to wild-type mice (p<0.01). In contrast, analysis of 35 E10.5 to E14.5 embryos from similar matings revealed 7 (20%) homozygotes, which is not significantly different from the expected ratio. These observations suggest that homozygosity for the *Pax2^A220G^* allele is lethal either later in gestation or perinatally. We have observed some *Pax2^A220G/A220G^* embryos, however, as late as E17.5 (n = 45).

**Figure 3:**
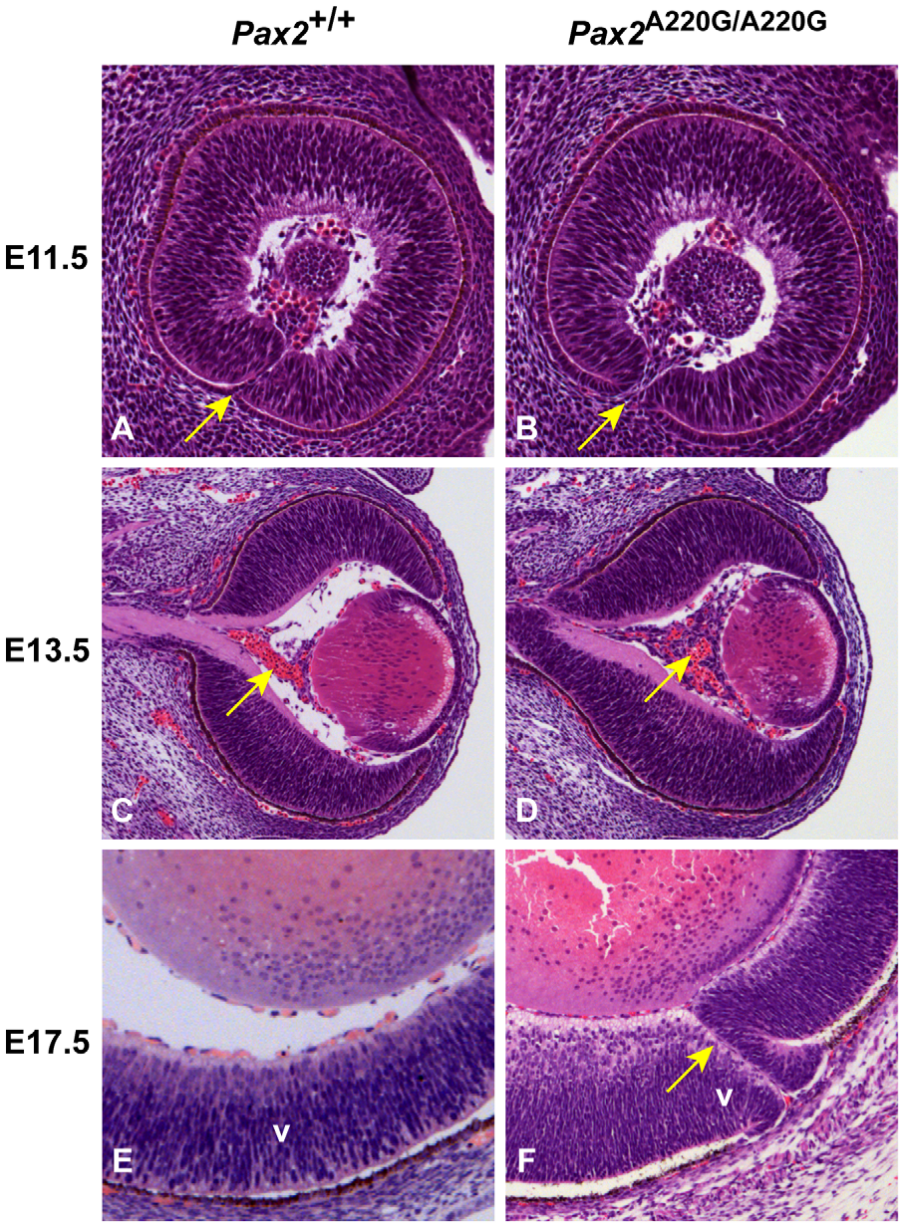
Histologic sections of *Pax2^+/+^* and *Pax2^A220G/A220G^* mouse eyes at three embryonic time points. At E11.5, parasagittal sections reveal a delay in apposition of the edges of the optic fissure in mutant mice (arrow) (A,B). At E13.5, coronal sections through the wild-type and homozygous mutant embryos reveal a delay in the formation of the *tunica vasculosis lentis* (arrow) (C,D). At E17.5, parasagittal sections demonstrate non-fusion of the optic fissure (uveal coloboma) in mutant embryos (arrow) (E,F). V = ventral retina.

Because *Pax2* null alleles had previously been reported to affect ocular, urogenital, and central nervous system development [22]–[25],[31], we examined these features pre- and postnatally in our mouse mutants. During ocular development in wild-type embryos, the edges of the optic fissure touch at E11.5 and fuse by E12.5 (Figure 3A and 3E). The invading mesenchyme has coalesced into a discernible central vascular trunk (the *tunica vasculosis lentis*) by E13.5 (Figure 3C). In contrast, *Pax2^A220G/A220G^* embryos have delayed optic fissure closure (Figure 3B), which sometimes results in frank uveal coloboma (Figure 3F). The differentiation of neural crest into discernible vascular structures is also delayed (Figure 3D). In addition to congenital optic nerve excavation, adult *Pax2^A220G/+^* mice exhibited variable, incomplete regression of the tunica vasculosis lentis, retinal dysplasia, bending of the retinal vasculature towards the dorsal retina, absence of a central retinal arterial trunk, and mild extension of the retinal pigment epithelium beyond the borders of the optic disc (Figure 1D, 1E, and 1G, and data not shown).

**Figure 4:**
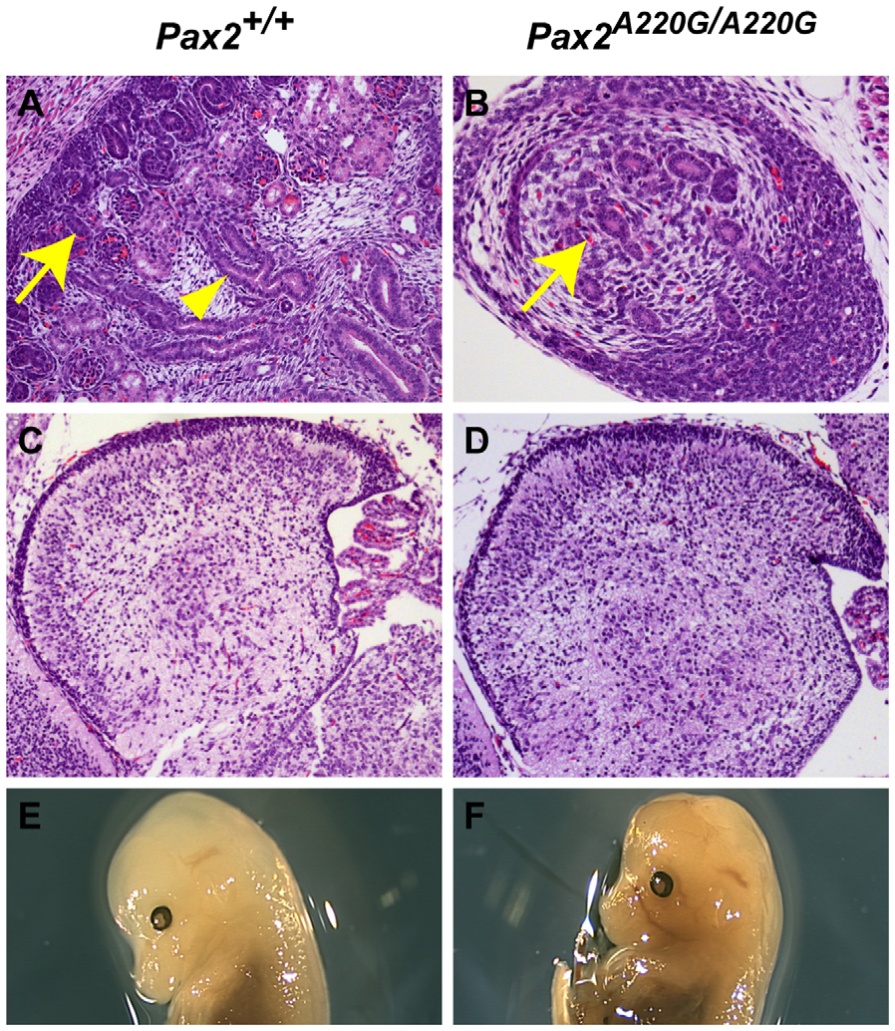
Histologic sections of *Pax2^+/+^* and *Pax2^A220G/A220G^* mouse kidneys (axial) and cerebellum (sagittal) at E17.5. Whereas wild-type mice have begun to develop renal glomeruli (arrow, A) and tubules (arrowhead, A), the mutant mice have only primordial kidneys with poor differentiation of these structures (arrow, B) In contrast, the differentiation of the cerebellum of both wild-type (C) and mutant (D) mice is comparable at this time, despite the midbrain-hindbrain boundary being a site of *Pax2* expression during embryogenesis. By E14.5, cranial structure was grossly normal in both wild-type (E) and homozygous mutant (F) embryos.

The developing kidneys of wild-type mice show induction of surrounding mesenchyme to form early glomeruli and tubules by E13.5 and have well differentiated cortical and medullary structures by E17.5 (Figure 4A). The kidneys of *Pax2^A220G/A220G^* embryos, however, show less induction of surrounding mesenchyme by E13.5, resulting in small, primordial kidneys at E17.5 (Figure 4B). Of the sixteen *Pax2^A220G/+^* mice (ages 1-4 months) analyzed with gross and microscopic pathology, 1/16 had bilateral cystic kidneys with hydronephrosis and hydroureter; 1/16 had unilateral renal hypoplasia with contralateral double papilla; 1/16 had unilateral renal hypoplasia with occasional focal cystic glomeruli; and 7 mice had bilateral, rare to occasional degenerative tubules on histologic sectioning. Of the seven age-matched, wild-type mice (14 kidneys) similarly examined, only one kidney had rare degenerative tubules on histologic sectioning.

In contrast to other *Pax2* mouse mutants [23],[24], the midbrain-hindbrain boundary of *Pax2^A220G/A220G^* develops relatively normally, as assessed by cerebellar development at E17.5 (n = 11 *Pax2^A220G/A220G^*, n = 12 *Pax2^+/A220G^* ) (Figure 4C and 4D). In embryos E10.5 to E12.5, 3/38 (8%) heterozygotes and 5/36 (14%) homozygotes had a mildly-flattened midbrain-hindbrain region, but otherwise normal isthmic structures. Cranial shape in homozygous mutant was grossly normal at E14.5 (Figure 4E and 4F) and at E17.5 (data not shown). We did not observe exencephaly, as has been previously reported (n = 45 wild-type, n = 45 heterozygote mutants, and n = 45 homozygotes) [23],[24]. Gross examination of the optic chiasm in homozygous mice showed no discernable abnormality (n = 45).

### Functional characterization of *Pax2* missense mutations

During our investigations, we noted that the predicted T74A mutation in mouse *Pax2* (which corresponds to T75 in the human protein), was next to or in the same location as two of the three missense mutations reported in humans—c.G226A (p.G76S) and c.220insGAGACC (p.74dupET) [15]. These mutations correspond to c.G223A (p.G75S) and c.222insGAGACC (p.73dupET), respectively, in the mouse sequence. For clarity, we will refer to these mutations using the mouse sequence, as this is the sequence that was experimentally tested.

As in the c.A220G (p.T74A) mutant, atomic modeling of the c.G223A (p.G75S) mutation shows that the hydrogen bond between nitrogen atom of glycine 75 and oxygen atom of tyrosine 71 located at 2.9 Å distance in the wild type Pax2 paired domain is broken (H-bond 3, Figure 2F). Both these mutations are affecting the conserved intramolecular interactions at the end of helix α3 and potentially could destabilize this local structure. In the c.222insGAGACC (p.dup73ET) mutation, the insertion of additional glutamic acid and threonine after amino acid 74 changes the local structure at the end of helix 3 and the beginning of the inter-domain linker (Figure 2E). This change causes a decrease in the secondary structure of the α3-helix and might introduce a conformational change in the inter-domain linker that could result in the loss of protein binding specificity.

**Figure 5:**
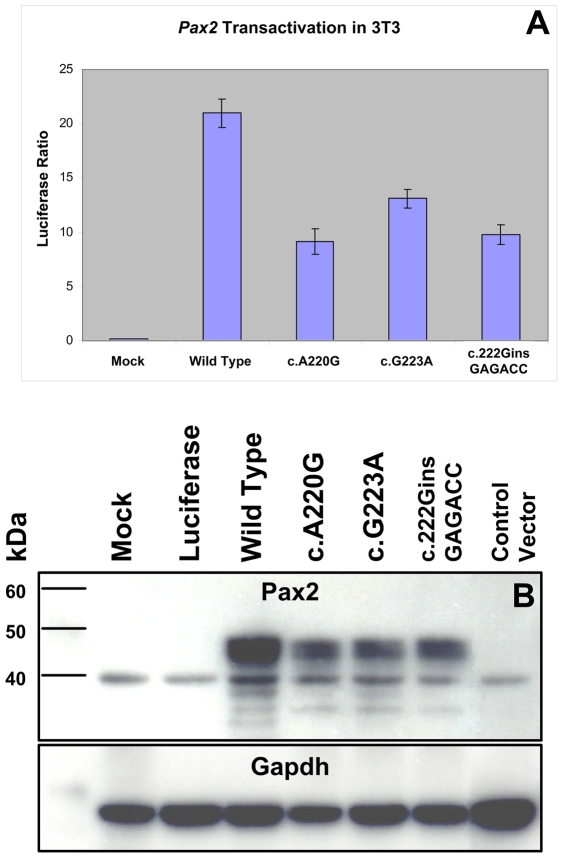
Comparison of wild-type and mutant Pax2 protein transactivation and expression in cell culture. NIH/3T3 cells were transfected with expression constructs for wild-type or mutant *Pax2* along with a *Pax2*-responsive luciferase reporter gene. All three mutants tested show reduced ability to transactivate (A). When steady-state levels of Pax2 protein were compared on Western blots from these experiments, mutants showed consistently lower levels of expression (B). Similar findings were observed when these experiments were replicated in COS-7 cells (data not shown).

**Figure 6:**
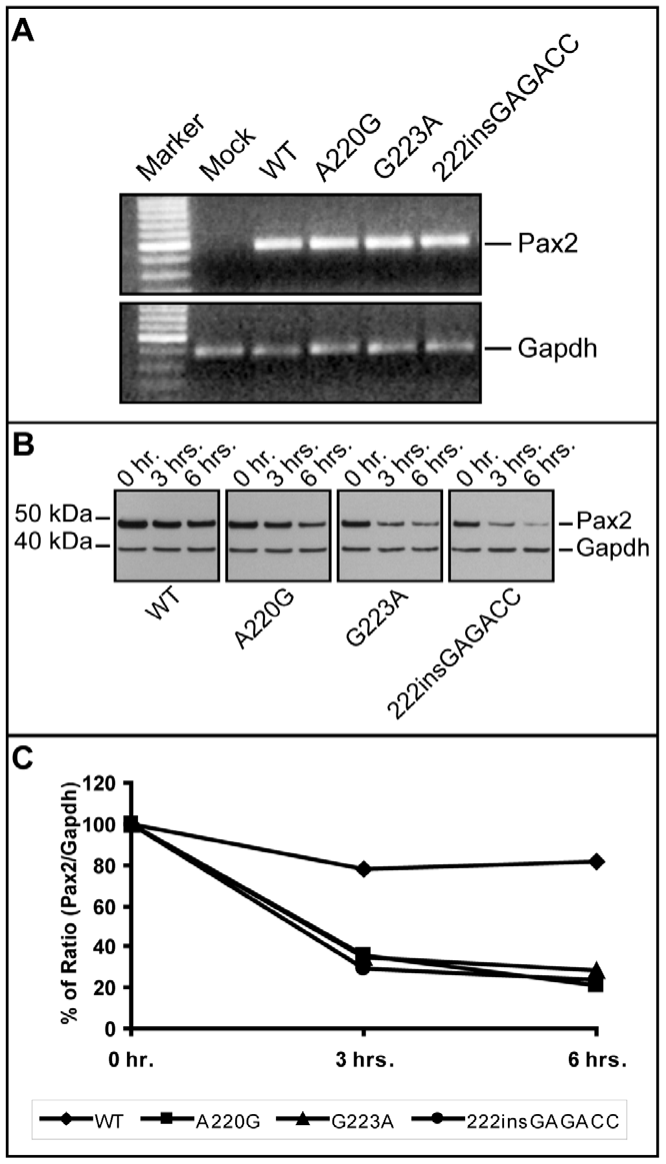
Comparison of *Pax2* mRNA steady-state levels and Pax2 protein stability in wild-type and mutant expression vector-transfected NIH/3T3 cells. Although steady-state levels of *Pax2* mRNA were comparable in wild-type and mutant transfected cells (A), the short-term protein stability of mutant Pax2 protein products were considerably reduced compared to wild-type, as determined in cycloheximide translation-inhibition experiments (B,C). See also Table 1 for quantification of mRNA levels.

In order to directly assay the effect these three missense mutations have on Pax2 function, we expressed them in a mouse fibroblast cell line (NIH/3T3) and compared their ability to drive expression of a Pax2-responsive reporter gene [32],[33]. All three mutant proteins showed an approximately 50% reduction in their ability to transactivate this reporter gene (Figure 5A). Reduced transactivation could be due to one or more of the following reasons: 1) reduced steady-state levels of *Pax2* mRNA, resulting in less Pax2 protein; 2) reduced stability of the abnormal Pax2 protein; 3) failure of the mutant Pax2 proteins to localize to the nucleus; and/or 4) failure of the protein to bind to DNA and transactivate target genes. In our cell culture model, all three mutant Pax2 proteins showed a considerable decrease in steady-state levels of protein expression when compared to wild-type protein (Figure 5B). Semi-quantitative image analysis of Pax2 band intensity demonstrated steady-state levels of 35% (*Pax2^A220G^*), 32% (*Pax2^G223A^*), and 38% (*Pax2^222insGAGACC^*) that of wild-type Pax2, when corrected for steady-state levels of Gapdh expression. Similar results were observed when the experiments were performed in COS-7 cells (data not shown). Steady-state levels of *Pax2* mRNA are similar in both wild-type and mutant construct-transfected cells (Figure 6A, Table 1), suggesting that our observations are mediated at the level of the Pax2 protein. In fact, the mutant Pax2 protein products are considerably less stable *in vitro–*as measured by a time-dependent decrease in Pax2 expression in cycloheximide-treated cells—than the wild-type protein (Figure 6B and 6C).

**Table 1:** Threshold cycle (C_t_) quantification of real-time, reverse-transcriptase PCR of wild-type and mutant *Pax2* transfected NIH/3T3 cells shows no significant difference in steady-state levels of *Pax2* mRNA relative to Gapdh.

Expression Vector	Pax2 (C_t_)	Gapdh (C_t_)
**Mock**	-	17.7
**WT**	26.1	15.7
**A220G**	23.9	15.0
**G223A**	23.9	15.2
**222insGAGACC**	23.3	15.5

**Figure 7:**
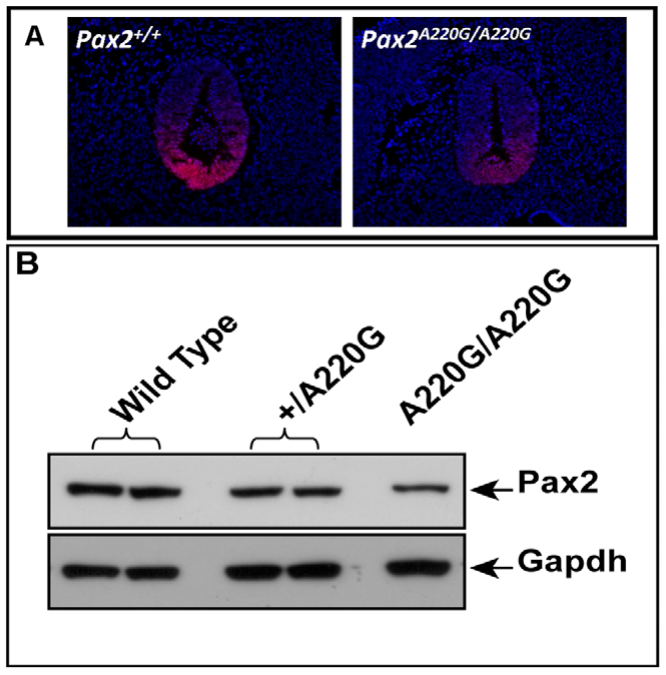
Comparison of wild-type and mutant Pax2 expression in embryonic mouse tissue. Pax2 immunofluorescence on parasagittal sections of E11.5 wild-type and homozygous mutant embryos demonstrate a normal pattern of expression in the ventral optic stalk (A). The level of Pax2 expression, however, is qualitatively reduced in the mutant mice. This reduced steady-state level of expression was confirmed by Western blot in heterozygous and homozygous mutant embryos (B).

**Figure 8:**
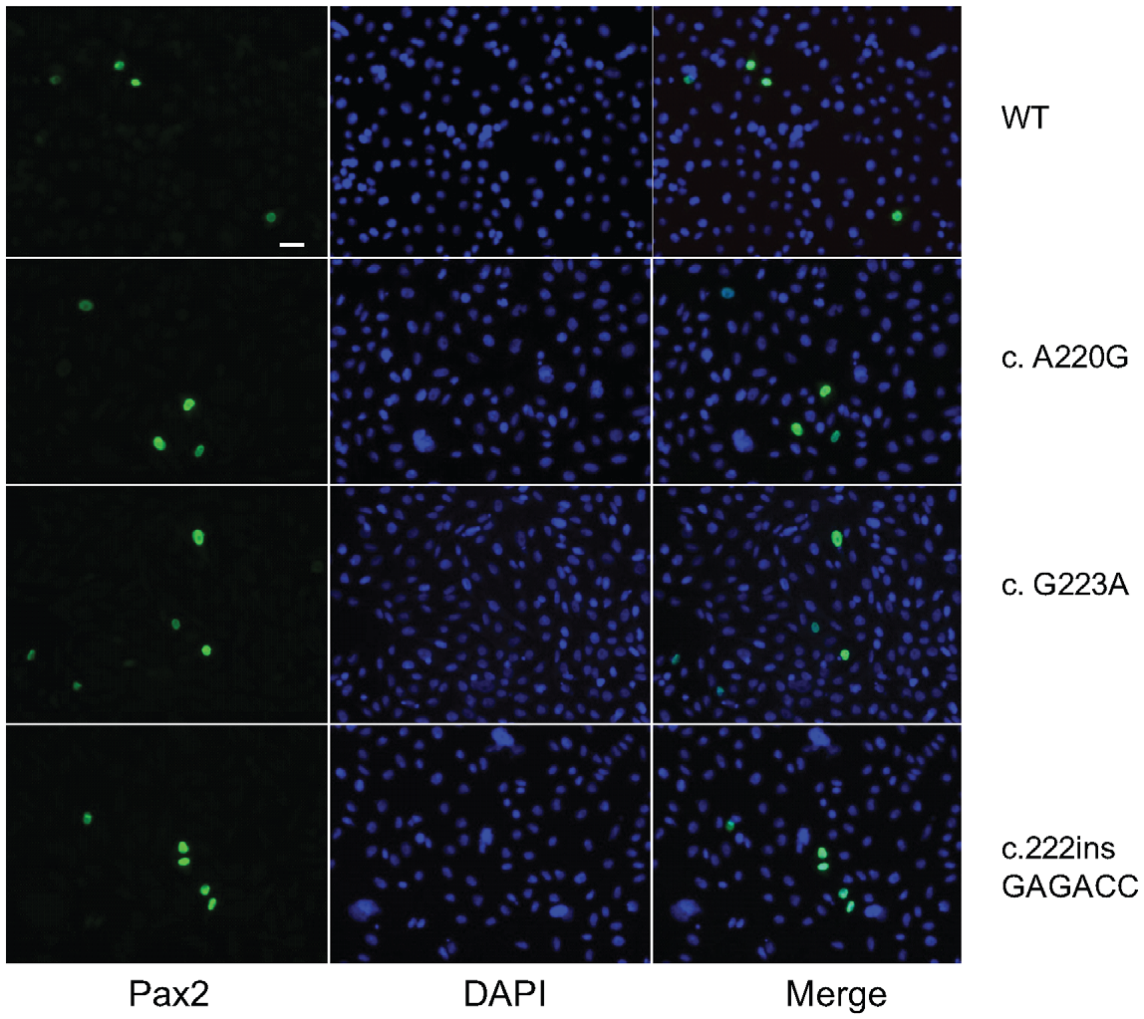
Pax2 immunofluoresence on COS-7 cells transfected with wild-type or mutant *Pax2* expression vectors. Both wild-type and mutant proteins display nuclear localization, as evidenced by co-localization of green fluorescence (Pax2) with DAPI nuclear staining (blue).

**Figure 9:**
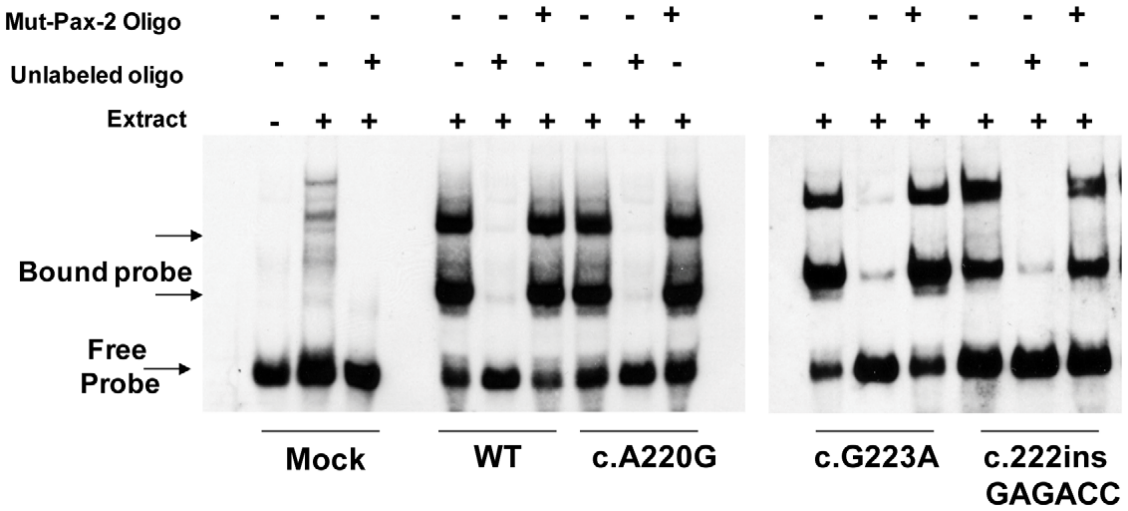
Electrophoretic mobility shift assay comparing DNA binding of wild-type and three mutant Pax2 proteins. A labeled Pax2 DNA-binding consensus sequence was incubated in the presence or absence of nuclear extract of COS-7 cells expressing equal amounts of the wild-type or mutant Pax2 protein; the same, unlabeled, competing DNA oligonucleotide; and/or a mutated version of the unlabeled oligonucleotide (Mut-Pax2). Nuclear extracts from mock transfected cells did not appreciably result in a shift of the labeled Pax2 DNA-binding site oligonucleotide, whereas wild-type and all three mutant Pax2 proteins bound the oligonucleotide with approximately equal affinity. Specificity for this binding was shown by competing the binding with the same, unlabeled oligonucleotide sequence and by failure of an unlabeled mutant oligonucleotide to compete for binding.

In support of this *in vitro* observation, the magnitude of Pax2 immunofluorescence was qualitatively reduced in the optic stalk of *Pax2^A220G/A220G^* E11.5 mouse embryos when compared to heterozygous or wild-type embryos (Figure 7A). (The overall expression pattern of Pax2 was, however, quite similar, making a gross patterning defect in the developing mouse eye less likely.) When relative levels of Pax2 expression were compared in head tissue from E11.5 mouse embryos by Western blot, a similar pattern was observed (Figure 7B). Semi-quantitative image analysis of Western blot band intensity demonstrated steady state levels of 54% and 13% in heterozygous and homozygous *Pax2* mutants, respectively, when compared to wild-type embryos, after corrected for Gapdh expression. Pax2 immunofluorescence on transfected COS cells demonstrated that wild-type and each of the three mutant proteins were uniformly, correctly targeted to the nucleus (Figure 8). As previously noted, this region of the Pax2 protein is not predicted to contact DNA and we therefore predicted that its ability to bind a Pax2 consensus sequence would not be drastically altered. In support of this *in silico* observation, electrophoretic mobility shift assays of the wild-type and mutant proteins showed no significant difference in their ability to bind a paired box consensus DNA sequence (Figure 9) at a concentration shown to be optimal for the wild-type protein. Taken together, these data suggest that the major pathophysiologic mechanism of these three missense mutations is to reduce the stability of Pax2/PAX2 protein and not to affect the steady state levels of *Pax2* mRNA, Pax2 protein localization or the ability of the protein to bind its DNA recognition sequence.

## Discussion

Although most mutations that cause PRS are predicted to cause complete loss-of-function of one *PAX2* allele, a few missense mutations clustering in the paired-box domain of the protein have been reported. This observation raises the possibility that a partially-functional or abnormally-functional protein product is made *in vivo*. We have identified a novel missense mutation in the mouse *Pax2* gene that is in the same position as one of the few human missense mutations.

Several lines of evidence suggest that this sequence change is pathological. This mutation absolutely co-segregates with an ocular and kidney phenotype reminiscent of human disease and of previously-reported mouse models of PRS [22]–[25]. The threonine residue affected by this mutation is absolutely conserved in all members of the paired-box family of transcription factors in mouse and is absolutely conserved in the *Pax2/PAX2* sequence across several species. Mutation at this residue has been described in individuals with PRS [15]. Furthermore, mutation of the corresponding threonine (T63P) in the human *PAX6* gene results in a relatively mild form of aniridia characterized by subtle iris hypoplasia, cataract and keratopathy along with nystagmus [34]. Lastly, our atomic modeling and *in vitro* functional studies suggest that this murine mutation, as well as the other human *PAX2* mutations reported in this region, mildly disrupt normal protein structure and result in hypomorphic alleles.

The mechanism by which *Pax2/PAX2* function is reduced in the three mutations tested is a reduced level of steady-state Pax2/PAX2 protein, likely due to a decrease in protein stability. Based on crystallographic evidence, this residue is not anticipated to contact DNA and electrophoretic mobility shift assays show no difference between the three mutant and wild-type proteins. These mutations do not affect the normal nuclear localization of the Pax2 protein *in vitro*. The protein is made *in vivo*, albeit at reduced levels. The reduction in transactivation observed *in vitro* approximates the reduction in steady-state protein levels *in vitro* or *in vivo* (for the c.A220G mutant mice). Reduced steady-state levels of protein have been proposed as a disease mechanism in other developmental eye diseases caused by mutations in transcription factor genes such as *FOXC1* and *PITX2* in Axenfeld-Rieger syndrome [35]–[37]. Interestingly, *increased* steady-state levels of another transcription factor protein, PAX6, are also thought to result in developmental eye disease (e.g., “partial aniridia”) [38], implying that perturbation of steady-state protein levels in either direction may cause disease. The relatively mild ocular presentation of the patients with missense mutations in this region of the PAX2 protein is also consistent with our finding that the three alleles tested are hypomorphic, rather than a complete loss-of-function [15]. However, we can not exclude the possibility that these missense mutations—which are translated into protein *in vitro* and *in vivo*—may also be affected Pax2/PAX2 interactions with other proteins in the transcriptional complex and that these abnormal protein-protein interactions are contributing to the pathogenesis of disease.

Our mouse model of PRS shares many phenotypic similarities to the two reported *Pax2* mutant models [22]–[24] and to the *Krd* mouse, which carries a large deletion on chromosome 19 that includes *Pax2* [25]. Similar to heterozygous, targeted *Pax2* knock-out mouse and the *Pax2^1Neu^* mouse [23],[24], *Pax2^A220G/+^* mice exhibit congenital excavation of the optic nerve head with extension of the retinal pigment epithelium beyond the optic disc. Although minor defects in retinal lamination similar to those described in the *Krd* mouse were noted in our model [25], we did not observe the gross thinning of the retina observed in the *Krd* mouse and the *Pax2^1Neu^* mouse. This difference may be attributable to the hypomorphic nature of our mutation, differences in background strain (e.g., the presence of a partial C3H background, which carries a mutation in phosphodiesterase that results in retinal degeneration [39]) and/or–in the case of the *Krd* mouse–the deletion of other genes in this region [40]. Unlike the directed knockout and the *Pax2^1Neu^* mouse [23],[24], we did not observe exencephaly or under-development of the midbrain-hindbrain region, as measured by cerebellar size and morphology. This difference may be due to the hypomorphic nature of our *Pax2* allele and/or differences in the background strain of mouse used. In fact, while Torres, et al. observed 11/59 exencephalic embryos on a mixed 129sv x NMRI background, they did not observe exencephaly in 14 homozygous knockout mice an inbred 129sv background [23]. The mild kidney phenotype seen in *Pax2^A220G/+^* mice and the severe phenotype observed in *Pax2^A220G/A220G^* mice are similar to those described in all three mouse models [22], [24]–[26]. Porteous and colleagues have previously shown that the renal hypoplasia seen in heterozygous *Pax2* mutant mice is likely due to increased apoptosis during fetal renal development [26].

The vascular patterning abnormalities that we observe in the *Pax2^A220G/+^* mice are notable, as they recapitulate the phenotype observed in patients with PRS, particularly the absence of a central retinal artery [7]. PAX2 is expressed in human astrocyte precursor cells and retinal astrocytes [41], which guide developing angioblasts during retinal vascular development [42]. Chu *et al.* have noted particularly strong Pax2 expression in astrocytes surrounding the optic nerve head, and suggest that the congenital optic nerve abnormalities noted in patients with PRS may be due to a deficiency of astrocytes [43]. Therefore both the congenital optic nerve excavation and the patterning abnormalities noted in the *Pax2^A220G/+^* mice may be due to a primary defect in astrocyte development and/or differentiation.

Threonine—the amino acid altered in the *Pax2^A220G/+^* mice—is a potential target for protein kinases. Phosphorylation of Pax2 by kinases such as the c-Jun N-terminal kinases (JNK) JNK-1 and JNK-2 enhances its ability to activate transcription [29],[30]. While most of this phosphorylation occurs in the serine/threonine-rich carboxyl terminus of the Pax2 protein, it is still possible that phosphorylation of the paired domain may regulate Pax2 transcriptional activity. However, our *in silico* analysis and our Western blot data do not suggest that threonine 74 (75 in humans) is a likely site of phosphorylation *in vitro* or *in vivo*.

Pax2, like all transcription factors, likely acts as part of a multi-protein complex to regulate transcription. For example, Gong *et al.* found that Pax2 forms a complex with Hox11 paralogous proteins and Eya1 and directly activates expression of *Six2* and *Gdnf* in the developing kidney [44]. While direct knock-out or nonsense mutation of Pax2 presumably abrogates all such interactions, the missense mutation of a well-conserved amino acid that results in an expressed protein provides the opportunity to ask more specific questions about how this area of Pax2 interacts with other proteins. We feel that our mouse model will enable us to begin to dissect physiologic Pax2 protein interactions and to help us better understand how disruption of such interactions leads to human disease.

## Materials and Methods

### Animal husbandry and clinical examination

C3H/HeJ (Stock # 000659) and C57BL/6J mice (Stock #000664) were obtained from The Jackson Laboratory (Bar Harbor, ME). Mice were housed according to our institutional Animal Review Board standards with a 14 hour light/10 hour dark cycle. These studies conformed to the principles for laboratory animal research outlined by the Animal Welfare Act (NIH/DHHS) and the ARVO Statement for the Use of Animals in Ophthalmic and Vision Research and were approved by the Institutional Animal Care and Use Committee of the University of California, Berkeley and the National Eye Institute. Ehtylnitrosourea (ENU) mutagenesis and breeding of mice were performed as previously described[45]. Briefly, male C57BL/6 mice were intraperitoneally injected with ENU (90 mg/kg body weight) weekly three times. Three months after the injection, each mouse was bred to wild-type C57BL/6J female mice to produce G1 mice, which were screened for dominant eye phenotypes. Clinical examination of the posterior segment was performed on gently restrained, awake mice after dilation with one drop of 1% tropicamide (Alcon Laboratories, Inc., Fort Worth, TX) using an indirect ophthalmoscope (Keeler, Windsor, Berkshire, UK) with a 90D condensing lens (Volk, Mentor, OH). The optic nerve phenotype was graded in each eye as follows: 0 = normal, +1 = mildly affected (anomalous nerve with peripapillary pigment changes), +2 = strongly affected (findings of “+1” and staphylomatous changes), or indeterminate. A mouse was deemed “affected” if it had a score of two or more for both eyes combined (i.e., at least a +1 score in each of both eyes or a +2 in one eye.) Mice were euthanized with carbon dioxide according to institutional guidelines. Enucleated adult mouse eyes were fixed in a phosphate-buffered paraformaldehyde-glutaraldehyde mixture according to published protocols [46]. Mouse embryos for histopathology were dissected on ice-cold phosphate buffered saline (PBS) and fixed overnight in phosphate-buffered 4% paraformaldehyde at 4°C. Hematoxylin and eosin-stained methacrylate sections via the pupillary-optic nerve axis (eyes) or in appropriate cross section (embryos) were used for histopathology.

### Genetic mapping

Microsatellite markers known to be informative for the two strains were chosen from the Mouse Mapping Primers v1.0 (Applied Biosystems, Foster City, CA). PCR conditions were as follows: 12 min denaturation at 95°C; 10 cycles of 94°C for 45 sec, 55°C for 1 min, and 72°C for 1 min; 20 cycles of 89°C for 1 min, 55°C for 1 min, and 72°C for 1 min; 10 min final extension at 72°C. The PCR products were pooled based on fluorescent labels and expected allele size. Fragment separation was achieved by capillary electrophoresis on a Genetic Analyzer 3100 using 36 cm capillary array and POP-4 polymer. The ROX400 size standard (Applied Biosystems, Foster City, CA) was run as an internal size-standard. Allele sizing was calculated using the local southern algorithm available in the GENESCAN software program (Applied Biosystems). Allele calling and binning was done using the GENOTYPER software (Applied Biosystems). All genotyping included control DNA from C57BL/6J strain, C3H/HeN strain and C57BL/6J x C3H/HeN.

### Modeling of PAX2 protein structure

The structure of wild-type Pax2 paired domain-DNA hetero-complex was modeled using the PAX6 paired domain-DNA complex structure (PDB: 6pax) from the RCSB database http://www.rcsb.org/pdb as the structural template [47]. Primary sequences of Pax2/PAX2 and Pax6/PAX6 from mice and human were aligned by the method of Needleman & Wunsch [48], and incorporated in the program Look, version 3.5.2 for 3-dimensional structure prediction [49],[50]. The wild-type Pax2 paired domain-DNA hetero-complex and mutation dup74ET were built by the automatic segment matching method in the program Look followed by 500 cycles of energy minimization [51]. The same program generated the conformation of the proteins with the T74A and G76S mutations; and refined them by self-consistent ensemble optimization (500 cycles) [50]. The geometry of the predicted structures was tested with the program Procheck [52].

### Immunofluorescence in cultured cells and mouse embryos

Mouse embryos were dissected in PBS and fixed overnight in 4% paraformaldehyde in PBS followed by cryopreservation in 30% sucrose overnight at 4°C. Whole embryos were embedded and frozen on dry ice in Neg –50 (Richard-Allan Scientific, Kalamazoo, MI). 10 µM frozen sections were cut through mouse eyes and slides were stored at −80°C. Tissue sections were then thawed and washed three times in PBS, and then treated with 1X target retrieval solution (pH 6) (Dako, Carpenteria, CA). After washing three times in PBS, the section was incubated in blocking reagent (10% normal donkey serum, 0.02% Triton X-100 in PBS) for 1 hr. at room temperature. Slides were incubated overnight in anti-murine Pax2 (1:1000, Covance, Berkeley, CA) at 4°C. Following removal of primary antibody slides were washed four times in PBS for ten minutes each and the Pax2 antibody was detected using Donkey anti-rabbit Alexa Fluor 594 secondary antibody (1:400, Molecular Probes Inc., Eugene, OR) for one hour at room temperature. Slides were then washed again in PBS and cover-slipped with Vectashield with DAPI (Vector Laboratories, Burlingame, CA). At least 100 cells were counted for each transfection.

For cell culture, COS-7 cells (ATCC, Manassas, VA) were cultured on slides and fixed in 4% paraformaldehyde at room temperature for 10 minutes, rinsed twice with wash buffer (0.1% Tween 20, 0.5% normal goat serum in PBS) then cryoprotected by incubation in 15% and 30% sucrose for 45 minutes each and stored at −80°C. When ready for use, slides were thawed, washed three times in PBS and incubated in blocking reagent (10% normal goat serum, 0.1% Tween 20 in PBS) for 30 min. at room temperature. Slides were incubated overnight in anti-murine Pax2 (1:200, Zymed, Carlsbad, CA) at 4°C. Following removal of primary antibody slides were washed four times in PBS for five minutes each and the Pax2 antibody was detected using goat anti-rabbit Alexa Fluor 488 secondary antibody (1:1000, Molecular Probes Inc., Eugene, OR) for one hour at room temperature. Slides were then washed again in PBS and cover-slipped with ProLong Gold with DAPI (Molecular Probes).

Fluorescence and brightfield images were taken with a Zeiss AxioVert 200 microscope with a digital camera connected to a PC running AxioVision 4.6.3 (Carl Zeiss MicroImaging, Thornwood, NY). When making qualitative comparisons of the intensity of immunofluorescence, care was taken to standardize exposure times.

### Lectin staining of retinal vasculature

Enucleated mice eyes were fixed in 4% paraformaldehyde in PBS at room temperature for 30 minutes. After washing in PBS, retinas were dissected and isolated. Four radial incisions were then made in preparation for flat mount. Subsequently, retinas were permeabilized and blocked in a solution of 0.5% Triton X100 and 1% bovine serum albumin in PBS at room temperature for 1 hour. After PBS wash, retinas were incubated in TRITC-conjugated lectin (1:100 dilution in PBS) overnight at 4°C. After rinsing, retinas were flat mounted with slow-fade medium (Pro-Long Gold, Invitrogen, Carlsbad, CA) and visualized under microscopy with a TRITC filter.

### Transactivation studies

PCR-based site directed mutagenesis of the CMV-*Pax2b* expression construct [32],[33] was performed according to standard protocol to introduce one of the following mutations: c.A220G, c.G223A, or c.222insGAGACC. These mutations in the mouse sequence correspond to c.A223G (p.T75A), c.G226A (p.G76S), and c.220insGAGACC (p.dup74ET) in the human sequence, respectively. Mutations were confirmed by direct sequencing. NIH/3T3 (mouse embryonic fibroblast) or COS-7 (African green monkey kidney) cells (ATCC, Manassas, VA) were plated at 0.25×10^6^ cells per well in a 6-well plate in DMEM media with 10% fetal bovine serum. The following day, cells were transiently transfected with 0.5 micrograms of PRS4-luciferase reporter construct [32],[33], 50 nanograms of *Renilla* luciferase construct (pRL-CMV, Promega, Inc., Madison, WI) and 2 micrograms of CMV-Pax2b expression construct (wild-type or mutant) [30] using 10 µl/well lipofectamine 2000 (Invitrogen, Carlsbad, CA) according to the manufacturer's protocol. Control samples were transfected with an equimolar amount of the expression vector backbone. After 48 hours, cells were harvested and luciferase activity was measured using microplate reader (Optima, BMG labtech, Durham, NC). All experiments were repeated at least three times with at least three replicates per sample.

### Western blotting

Transfected cells were harvested for protein in 1x Passive Lysis Buffer. Total protein was determined using Micro-Lowry method (Sigma-Aldrich, St. Louis, MO). Equal amounts of protein were separated (4–12% NuPAGE Bis-Tris) polyacrylamide gels and transferred to 0.2 µm, PVDF membranes (Invitrogen, Carlsbad, CA). Blots were hybridized with 1:1000 dilution Rabbit anti-Pax2 antibody (Zymed, San Francisco, CA) and 1:4000 dilution of goat anti-rabbit-HRP secondary antibody (Thermo Fischer Scientific, Pierce Protein Research Products, Rockford, IL) and then developed with SuperSignal West Pico chemiluminescent substrate for detection of HRP (Thermo Fischer Scientific, PierceProtein Research Products, Rockford, IL). Quantitation was performed on a ChemiDoc EQ (Bio-Rad Laboratories, Hercules, CA) using the manufacturer's software (Quantity One, v.4.5.2, Build 070).

### Quantification of wild-type and mutant *Pax2* mRNA levels

NIH/3T3 cells were transiently transfected with wild type or mutant Pax2 plasmids using FuGene 6 HD reagent (Roche-Applied Science, Indianapolis, IN). 48 hrs. post transfection, RNA was isolated using an RNeasy mini kit from Qiagen. 1 µg of RNA was treated with DNase I to remove any DNA contamination from the samples. cDNA was prepared using High Capacity cDNA Reverse Transcription kit (Applied Biosystems, Foster City, CA). Real time PCR was performed with SYBR green master mix (Applied Biosystems) on Bio-Rad iCycler. The following primers were used for Pax2 (Forward- TATGCACTGCAAAGCAGACC and Reverse- GGGGCAGTCACTCCTGTC) and Gapdh (Forward- GCATTGTGGAAGGGCTCATGACC and Reverse-CGGCATCGAAGGTGGAAGAGTGG). The parameters for PCR amplification were 95°C for 10 min followed by 40 cycles of 95°C for 30 s, 55°C for 30 s and 72°C for30 s. Relative expression of different mRNA samples for *Pax2* and *Gapdh* was calculated using the comparative threshold cycle method.

### Protein degradation analysis

Pax2 wild type and mutant proteins stability were characterized by transiently transfecting NIH/3T3 cells with expression vectors expressing wild type and mutant Pax2 proteins using a modified method described by Jiang et al [53]. In brief, 24 hrs post-transfection with equal amounts of wild-type or mutant *Pax2* expression vector, cells were treated with cycloheximide (CHX) (100 µg/ml) (Sigma-Aldrich, St. Louis, MO). Cells were washed with PBS and lysed with RIPA buffer at 0, 3 and 6 hrs after CHX treatment. The protein concentrations in the lysates were determined by BCA method (ThermoFisher Scientific-Pierce, Rockford, IL). Equal amounts of proteins were resolved in NuPAGE Novex Bis-Tris gel (4–12%), transferred to PVDF membrane and probed with Rabbit polyclonal anti-Pax2 (1:250 dilution) and anti-Gapdh (1:500 dilution) primary antibodies. HRP conjugated anti-rabbit IgG was used as secondary antibody (1:5000 dilution). SuperSignal West Pico Chemiluminescent Substrate was used to detect HRP on the blots. The blot was imaged using Autochemie System (UVP, Upland, CA) and quantitative analysis of the bands was performed using Labworks software.

### Gel mobility shift assays

COS-7 cells were transiently transfected with CMV-Pax2a expression construct (wild type or mutant) using FuGENE 6 HD reagent (Roche-Applied Science, Indianapolis, IN). The cells were harvested after 48 h and the nuclear extracts were prepared by using NE-PER nuclear and cytoplasmic extraction kit reagents (Thermo Scientific-Pierce, Rockford, IL). Gel purified sense and antisense oligonucleotides representing wild-type or mutant Pax2 DNA binding sites were labeled at the 3′ end of DNA strand with Biotin -11-dUTP (Biotin 3′ end DNA labeling kit, Thermo Scientific-Pierce). The forward wild-type primer was 5′TGGAATTCAGGAAAAATTGTCACGCATGAGTGGTTAGCTCGAGTA-3′ and the forward mutant primer was 5′TGGAATTCAGGAAAAATTTGATACCATGAGTGGTTAGCTCGAGTA-3′, where the underlined sequence represents the portion of oligonucleotide that was mutated. Gel mobility shift assays were performed with Biotin -11-dUTP labeled target DNA in 10 mM Tris-HCl pH 7.5, 100 mM KCl, 0.5 mM DTT, 05% NP-40, 2.5% glycerol and 50 ng/µl poly (dI:dC) and incubated with the Wt or Mut nuclear extracts in a total volume of 20 µl. After incubation for 20 minutes, protein-DNA complexes were separated on a 6% polyacrylamide gel in 0.5 x TBE buffer at 100 V/cm, transferred onto Nytran membrane and UV cross-linked. Biotin label was detected by chemiluminescent detection module (Thermo Scientific-Pierce) that uses luminol substrate for HRP-conjugated streptavidin. For competition experiments, the nuclear extracts were pre-incubated with 100 molar excess of unlabeled or mutant DNA for 10 min. before adding the biotin labeled probe.
